# Exploring the spatial coupling relationship between green vegetation carbon stock and recreational intensity in urban parks: A case study of Hangzhou

**DOI:** 10.1371/journal.pone.0345031

**Published:** 2026-03-30

**Authors:** Qingqing Huang, Anhua Qin, Alin Lin, Zongjie Liu

**Affiliations:** 1 School of Civil Engineering and Architecture, Zhejiang Sci-Tech University, Hangzhou, Zhejiang, China; 2 College of Design and Engineering, National University of Singapore, Singapore, Singapore; 3 Linyi Data and Application Center of the National High-Resolution Earth Observation System, Linyi, Shandong, China; Zhejiang Agriculture and Forestry University: Zhejiang A and F University, CHINA

## Abstract

As an important form of green space within densely built urban environments, optimizing the layout of city parks based on the synergistic integration of carbon stock and recreational services level holds significant importance. Taking Hangzhou Jinsha Lake Park as a case, this research employs remote sensing inversion to analyze the spatial distribution of the park’s carbon stock. A set of indexes was used to evaluate the park’s recreational services level. Finally, a coupling coordination model was applied to generate and visualize the coupling coordination degree between the park’s carbon stock and recreational services level. Results indicate that the coupling coordination degree between recreational services level and carbon stock in Jinsha Lake Park exhibits a distribution pattern characterized by higher values in the west and lower values in the east, as well as higher values in the south and lower values in the north. Most of these coupling coordination degrees fall in the “primary coordination” range and exhibit different spatial characteristics, such as “high recreational level – low carbon stock” and “low recreational level – high carbon stock”. Park road accessibility serves as a fundamental prerequisite for activating spaces. The rational allocation of functional zones provides a structural framework for balancing ecological and recreational interests, while optimized vegetation configuration acts as the core engine that directly drives the simultaneous growth of carbon stock and recreational vitality. Appropriate plant configuration not only directly increases regional carbon stock but also boosts recreational vitality by increasing plant diversity and vegetation coverage. This study proposes a park optimization strategy based on the synergy between carbon stock and recreational functions, which is of great significance for advancing the dual objectives of enhancing ecological services and social services in urban parks.

## Introduction

Rapid urbanization has triggered a sharp rise in energy consumption and carbon emissions [1]. Currently, urban areas account for 67%-76% of global energy consumption and generate 71%-76% carbon dioxide emissions [2]. The reduction in energy consumption and the enhancement of carbon sink capacity in cities are the key to achieving global decarbonization goals [3].

Urban parks play significant roles in microclimate regulation [4], noise reduction [5], storm runoff interception, and air purification [6]. Additionally, urban parks can enhance carbon sink capacity and oxygen release, playing a vital role in reducing urban carbon emissions [7]. A rational layout and design of urban parks not only boosts the carbon sink capacity of urban green spaces but also effectively promotes recreational activities and physical exercise among residents. This significantly contributes to their physical and mental health as well as their subjective well-being [8]. Therefore, the synergistic development of the ecological and social services of urban parks represents an essential pathway to enhance urban sustainability.

The current studies on urban green space carbon sinks have concentrated on different spatial levels. At the macro level, studies revealed the spatial distribution characteristics of carbon stock in urban green spaces and estimated carbon stock at the city scale [9,10]. At the meso level, most studies have focused on national parks or large nature reserves to investigate carbon stock [11] and their influencing factors. Moon et al. demonstrated that decreased lawn area, increased broad-leaved trees, and reduced lawn management intensity are the most effective approaches to enhancing a park’s carbon stock [12]. Li et al. studied riparian spaces and found that the internal green component factors (including vegetation coverage and tree green ratio) are key factors influencing carbon stock [13]. At the micro level, research has deepened into plant communities and tree species, focusing on identifying the carbon sequestration capacity and oxygen release capacities of specific planting species and their configurations [14,15]. Meanwhile, with the development of science and technology, a number of computer model software programs have been successively developed to calculate vegetation carbon stock or carbon sequestration benefits, such as CITY green, i-Tree, etc. Additionally, remote sensing and GIS technologies have also become increasingly mature in the research and application of urban green space carbon stock. Cheng et al. used multi-source data including remote sensing to estimate the above-ground biomass (AGB) of forests [16]. Oehmcke et al. used remote sensing with airborne LiDAR to measure vegetation structure on a large scale. Therefore, maximizing carbon stock benefits within the constrained green space of city parks has emerged as a critical research topic [17].

Urban parks serve as one of the most frequently visited locations for residents’ daily leisure activities. Their recreational services provide citizens with spaces to connect with nature and relax, acting as a vital means to enhance public well-being and life satisfaction. To comprehensively analyze the effectiveness of park recreation services, existing research has established a multidimensional indicator framework encompassing supply, demand, and perception dimensions. This framework integrates diverse methodologies such as questionnaire surveys, social media big data analysis, and GIS spatial observation. For instance, Zhang et al. evaluated park recreation services using indicators like aesthetic features, facilities for recreation, and facilities for convenience [18]. Hamstead et al. employed geolocated social media methods to explore changes in visitation across 2,143 different parks in New York City [19]. This multifaceted exploration of park recreational services level guides us in scientifically planning how green spaces can be transformed into key elements for enhancing urban vitality, resident well-being, and sustainable development capacity.

There are limitations in focusing solely on maximizing carbon stock or recreational services level in urban parks. As a complex ecosystem providing both ecological and social services, the quality of a city park depends on the balance between its internal ecological elements and recreational functions. The coupling coordination model refers to the synergistic state formed by mutual constraints and connections between multiple systems or among elements within a system, driven by certain interactions or responses. It is currently widely applied to quantitatively assess the intensity of interactions and synergistic effects among various elements within parks. For instance, Othman et al. investigated how plant material specifications and spatial design patterns in urban parks influence carbon stock, finding that specific design forms combined with particular plant material can achieve higher carbon sequestration potential [20]. Liu et al. assessed the coupled coordination between ecological connectivity and spatial accessibility in urban green spaces, revealing that over 70% of urban green spaces exhibit imbalances [21]. However, due to the current lack of research on the synergistic mechanisms between carbon stock and recreational services level in urban parks, there is insufficient empirical understanding of their interaction mechanisms and coupling relationships, which directly leads to a lack of necessary scientific guidance for park planning and management. To address this issue, this paper takes Hangzhou Jinsha Lake Park as an empirical case study and introduces a coupling coordination model. It focuses on investigating the interaction intensity and coordinated development level between urban parks’ carbon stock and recreational functions. Through this research, we aim to support the sustainable development of urban parks’ multiple functions. The primary research objectives are as follows: (1) Establish a comprehensive evaluation index system for recreational services level. (2) Reveal the coupled coordination relationship between the park’s carbon stock and recreational services level from the perspective of functional zones. (3) Based on empirical findings, propose spatial planning and adaptive management strategies aimed at promoting synergistic enhancement of the park’s carbon stock and recreational functions.

## Materials and methods

### Study area

**Fig 1:**
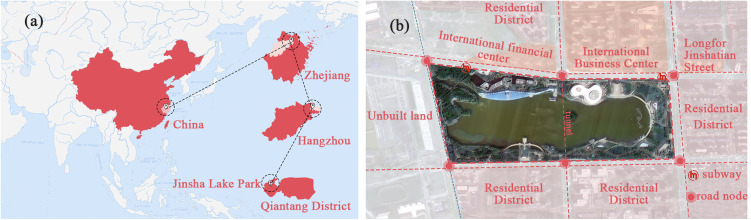
(a) Location of the study area in China. The map shows its location within the nested administrative boundaries of China, Zhejiang Province, Hangzhou City, and Qiantang District. (b)The surrounding environment of Jinsha Lake Park. The adjacent land is primarily occupied by residential and commercial zones. The park is situated near two subway stations to the north and is flanked by major urban roads to the north and south, indicating good transport accessibility and a favorable location.Reprinted from Linyi Data and Application Center of the National High-Resolution Earth Observation System under a CC BY license, with permission from Zongjie Liu, original copyright 2024.

This study focuses on Hangzhou Jinsha Lake Park (120°19′11.68″E, 30°18′27.65″N) in China, covering a total area of 64 hm², with water bodies occupying nearly 27 hm² (Fig 1). This study aims to explore the interaction intensity and level of coordinated development between its carbon stock and recreational services level. The reasons for selecting Hangzhou Jinsha Lake Park as the study site are as follows: (1) Located in the core area of Hangzhou’s Qiantang District, Jinsha Lake Park is surrounded by mature commercial and residential zones. As a new park built in the past decade, it has modern and comprehensive recreational facilities. These multiple advantages have made Jinsha Lake Park’s recreational services level exhibit the typical characteristics of high intensity and diverse complexity.(2) Jinsha Lake Park employs a typical zoned planting strategy, featuring diverse areas such as dense woodlands preserving existing large trees and open lawn zones prioritizing recreation. This creates conditions for conducting comparative studies within the same park. In summary, the recreational vitality derived from Jinsha Lake Park’s advantageous geographical conditions, along with its own distinctive zoned planting features, makes it an exemplary case study for examining the carbon stock and recreational services level of urban parks.

This study mainly focuses on six typical functional zones of Jinsha Lake Park, including the entrance distribution areas (north and south main entrances, and northwest and southeast secondary entrances), vibrant waterfront zone, waterfront leisure area, woodland recreation areas (southeastern and northwestern ones), children’s activity zone, and tranquil relaxation zone. It explores the significant differences in coupling coordination levels among the park’s functional zones (especially zones of the same type) and the causes of their spatial heterogeneity, and proposes improvement strategies for the existing park.

### Data analyzing procedures

**Fig 2:**
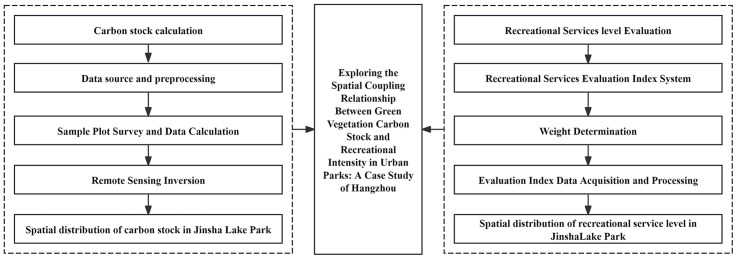
Research framework. The flowchart illustrates the three core steps of the study: carbon stock simulation (left), recreational service evaluation (right), and their coupling coordination analysis (center).

The workflow of this study is shown in Fig 2, which mainly consists of three steps: (1) The simulation of carbon stock. Based on the on-site investigation data, a remote sensing inversion model was established using high-resolution satellite images to simulate the spatial distribution of carbon stock. (2) The evaluation of a park’s recreational services level. This study developed an evaluation system to measure the recreational services level of a park by using the Analytic Hierarchy Process (AHP) method. (3) A coupling coordination model was employed to expose the synergistic relationship between the park’s carbon stock and recreational services level. The following will provide a detailed description of the process.

### Carbon stock estimation

The vegetation carbon stock consists of both above-ground biomass and below-ground biomass, and previous research indicates that above-ground biomass constitutes the primary component [22]. Therefore, this study excludes carbon pools from the below-ground vegetation components, such as dead wood, leaf litter, and soil layers, from carbon stock calculations. Estimates are only based on the living biomass of the aboveground parts of individual trees.

(1) **Data sources and preprocessing.** The original satellite remote sensing image data were provided by the Linyi Data and Application Center of the National High-Resolution Earth Observation System, sourced from the GF-1 satellite of the China Platform of Earth Observation System. The selected image was acquired on April 18, 2024, with cloud coverage below 10% and a spatial resolution of 2 meters. A series of processing steps were subsequently applied to the raw data using professional software such as PIESAT PIE-Ortho 6.3.
(2) **The extraction of NDVI.** The Normalized Difference Vegetation Index (NDVI) is widely used to determine carbon stock in above-ground biomass [23]. NDVI is calculated from remote sensing data using the near-infrared and red bands as follows:

$$\begin{array}{c} NDVI=NIR-RED/NIR+RED) \end{array}$$ (1)

NIR: Near-infrared band reflectance; RED: Red band reflectance.

**Fig 3:**
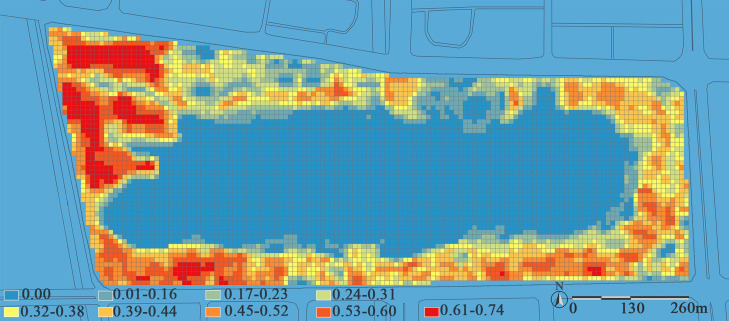
NDVI distribution map of Jinsha Lake Park. The NDVI values in the figure range from 0.00 to 0.74 and are divided into nine levels. A blue-to-red color gradient is used for visualization, where redder hues indicate higher NDVI values, corresponding to denser vegetation and greater biomass; bluer hues represent lower NDVI values, associated with water bodies or sparse vegetation.

The NDVI values range from -1.0 to 1.0. When the value is below 0.1, the ground surface has almost no vegetation. To mitigate the impact of NDVI variations in bare ground on the model, all NDVI values within the range [-1.0, 0.1] are set to 0. As shown in Fig 3, the NDVI values of Jinsha Lake Park exhibit significant spatial heterogeneity, specifically manifesting as a distribution pattern characterized by “higher values in the west and lower in the east, higher in the south and lower in the north”. This pattern results from the sharp contrast between the densely vegetated western native forest and the heavily built-up northern region, which is dominated by impervious surfaces. The limited greenery there, such as rooftop gardens, does little to boost overall vegetation coverage.

**Fig 4:**
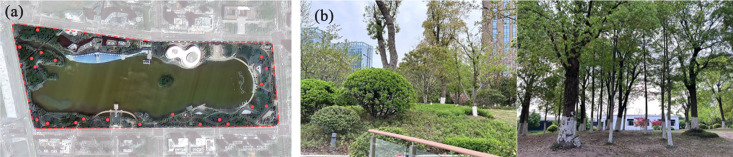
(a) The distribution of sampling plots. The red circles indicate the center points of the 10 m × 10 m sample plots. Reprinted from Linyi Data and Application Center of the National High-Resolution Earth Observation System under a CC BY license, with permission from Zongjie Liu, original copyright 2024. (b) Field Photographs from Sample Plots. These images depict representative vegetation conditions within the surveyed sample plots at Jinsha Lake Park.

(3) **Sample extraction and carbon stock estimate.** This study conducted plot surveys in April 2025, which lasted one month. The specific procedures are as follows: The sample plot size is 10 × 10 meters [24]. First, using stratified random sampling, 23 sample plots were selected within the study area. After excluding water bodies and buildings, the “Create Random Points” tool in ArcGIS 10.6 was used to get the coordinates for each sample plot. Second, we adjusted the selected sample plots in the first step through field surveys to ensure the sample plots include diverse vegetation types (tree-shrub-herb and shrub-herb). Third, data on plant species, tree height, and diameter at breast height (DBH) were recorded during the on-site investigation. Finally, 21 sample plots and their detailed data were obtained (Fig 4).

After the on-site investigation, we used the collected data to estimate the carbon stock using the biomass equation method. Biomass equations for urban trees were selected from Chinese literature datasets and referenced relevant equations compiled by Wang C, Hyun-kil, and HE H Z. (Table 1). If no applicable biomass equation existed for a specific tree species, equations the same family or genus, or a generalized biomass equation for broadleaf trees or coniferous trees were applied to estimate vegetation carbon stocks.

**Table 1:** Biomass equations for different tree species.

Number	Plants	Biomass Equation	Source
1	*Cinnamomum camphora*	*W* = 0.937 + 0.037*D*^2^*H*	HE et al., 2007 [25]
2	*Koelreuteria paniculata*	*W* = 0.915 + 0.1*D*^2^*H*	
4	*Acer* spp.	*W* = 0.1766*D*^2.381^	Wang C. 2006 [26]
5	*Salix babylonica*	*W* = 0.1633*D*^2.417^	
6	Coniferous Trees General	ln*W* = −2.2796 + 2.2874ln*D*	Hyun-kil, Jo 2002 [27]
7	Broadleaf Tree General	ln*W* = −3.5618 + 2.6645ln*D*	

*W* represents above-ground biomass.

*D* represents diameter at breast height (DBH).

*H* represents tree height.

(4) **Remote sensing inversion.** This study estimates the spatial distribution of carbon stocks across Jinsha Lake Park by constructing a remote sensing inversion model based on plot-level carbon stock estimates. First, Pearson correlation analysis was used to investigate the relationship between carbon stock and NDVI of sample plots by using SPSS software [28]. The correlation coefficient was 0.656 with a *p*-value of 0.006, indicating a significant relationship between the two variables. Second, we constructed a regression model to fit the relationship between carbon stock and NDVI of sample plots. The data were divided into two parts: 70% of the sample data was randomly selected as the dependent variable for model construction. The model was Y = 39.842X^1.420^, with R^2^ = 0.690, F = 31.143, with *p* < 0.01. We substituted the remaining 30% data into this formula to validate it, yielding an RMSE of 1.362 and an MRE of 8.09%. The deviation between predicted and actual values falls within an acceptable range, confirming that the model can be used for remote sensing inversion.

### The evaluation of recreational services level

(1) Recreation indicator screening.

#### Indicator construction.

Park recreation services level directly reflects the comprehensive capacity to provide residents with leisure,activity, and ecological experiences. To ensure the scientific evaluation of the recreation services level, this study prioritized quantifiable objective data in the evaluation indicator system based on a literature review. Previous studies have indicated that the fundamental attractiveness and frequency of use of urban parks are significantly and positively influenced by factors such as road network accessibility, adequate service facilities, and water body distribution [29]. At the same time, plant diversity within parks has been shown to be closely associated with users’ psychological restorative benefits and positive perceptions [30]. Furthermore, some scholars have pointed out that facility density, the proportion of tree canopy-covered areas, and the richness of land cover types are all crucial for recreational activities in urban parks [31]. Building on this, a preliminary selection pool consisting of 11 indicators has been constructed for the Park recreation service level evaluation index system, ensuring that our screening process is grounded in solid theoretical foundations and prior research.

#### Indicator screening.

In the indicator screening phase, our team formed an expert panel with invited professors and senior planners in the field. Centered on the core objective of “carbon stock-recreational services level synergy,” we conducted multiple rounds of discussion and evaluation on the initially selected indicators. To ensure the scientific rigor and practicality of the Park recreation services level evaluation index system, we established the following three screening principles: ① Goal Relevance: Indicators must reflect the synergistic relationship between carbon stock and recreational functions, avoiding deviation from the theme. ② Independence: Each indicator should possess clear informational distinctiveness to avoid overlap and redundancy. ③ Data Availability and Quantifiability: Indicators should be obtainable through methods such as remote sensing, field surveys, or questionnaires, and should support subsequent spatial analysis and visualization. After repeated discussions, the expert panel concluded that the two indicators, “Plant Hierarchical Structure” and “Spatial Comfort,” were relatively insufficient in terms of data availability and indicator uniqueness, and therefore were not included in the final Park recreation services level evaluation index system. Ultimately, we established nine key indicators-Road network accessibility, Service facilities, Buildings and structures distribution, Functional diversity, Plant diversity, Vegetation coverage, Waterbody distribution, Waterbody morphological diversity, and Micro-topography shaping-that align with the research objective, are mutually complementary, and balance scientific validity with operational feasibility. This formed the Park recreation services level evaluation index system, which is a clear, hierarchical, and purpose-oriented framework, laying a solid foundation for subsequent in-depth quantitative analysis and planning practice.

(2) Weight determination.

The Analytic Hierarchy Process (AHP) was employed to determine the weights of each recreation services level indicator. AHP is a widely used method in multi-attribute decision-making [32]. It decomposes complex problems into multiple hierarchical levels, enabling the quantification of subjective judgments and qualitative analysis, thereby expressing them in an objective manner [33].

#### Questionnaire design and distribution.

To ensure the scientific validity and professionalism of the weight assignments, we formed an expert panel comprising 18 professionals with extensive experience in the fields of landscape architecture, ecological planning, and recreational studies. Based on the constructed Park recreation services level evaluation index system, we used a professional online analysis platform, SPSSPRO, to create the AHP expert consultation questionnaire. The questionnaire consisted of three parts:① Explanations of the AHP pairwise comparison matrix and definitions of the Fundamental Scale of 1-9 (Table 2). ② Specific definitions and explanations of each indicator to ensure a consistent understanding among the experts. ③ A request that the experts conduct pairwise importance comparisons-first among the dimensions under the criterion layer and then among the specific indicators within the same dimension-by completing judgment matrices. The questionnaires were distributed to the aforementioned experts via targeted emails and offline academic conferences. A total of 18 questionnaires were collected, resulting in a 100% response rate.

**Table 2:** The Fundamental Scale of 1-9.

Scale	Definition (comparing Factor i with Factor j)
1	Factor i is of equal importance to Factor j
3	Factor i is slightly more important than Factor j
5	Factor i is moderately more important than Factor j
7	Factor i is strongly more important than Factor j
9	Factor i is absolutely more important than Factor j
2,4,6,8	Between the two adjacent situations above
Reciprocal	Compare the two factors in reverse

#### Data processing.

After collecting the questionnaires,we used the online analysis platform SPSSPRO was used to calculate the Consistency Ratio (CR) for each questionnaire to ensure the scientific validity and reliability of the Analytic Hierarchy Process (AHP) results. This step is necessary because the AHP method, which employs the Fundamental Scale of 1-9, is based on experts’ pairwise comparisons of indicators. As these comparisons represent subjective judgments, logical inconsistencies may arise in multiple evaluations. According to AHP standards, a judgment matrix is considered acceptably consistent when its CR value is less than 0.1. If any judgment matrix from an expert had a CR value≥0.1, all judgments from that expert were deemed to lack logical consistency and were therefore excluded entirely. Ultimately, all judgment matrices from 15 experts met the consistency requirement (CR＜0.1), and their questionnaires were validated as effective. Subsequently, SPSSPRO was used to calculate the criterion-level indicator weights corresponding to each expert’s judgment matrices. These weights were then aggregated using the geometric mean method to derive the final weights for each recreational services level indicator (Table 3).

**Table 3:** Park Recreational Services Level Evaluation Index System.

1st Layer	Weight	2nd Layer	Weight	Data Sources
Spatial design	0.563	Road network accessibility	0.162	Satellite image
		Service facilities	0.157	Field research
			Buildings and structures distribution	0.080
		Functional diversity	0.164	Planning document
Natural ecology	0.437	Plant diversity	0.123	Field research
		Vegetation coverage	0.137	Satellite image
			Waterbody distribution	0.079
			Waterbody morphological diversity	0.057
		Micro-topography shaping	0.041	Planning document

(3) The recreational services level evaluation of Jinsha Lake Park.

A 10 × 10 m grid was established in ArcGIS 10.6. Data for service facilities allocation, distribution of buildings and structures, and plant diversity were collected through field surveys. By recording the coordinates of facilities such as benches, trash bins, and pergolas, their spatial locations were mapped onto the grid, allowing for visual distribution mapping. Data on road network accessibility, vegetation coverage, waterbody morphological diversity, and waterbody distribution were derived from satellite image interpretation. For instance, road network accessibility was assessed by calculating road density within each grid to evaluate transportation convenience.

For predicting the spatial distribution of plant diversity, many scholars have adopted the Ordinary Kriging interpolation method to predict the spatial distribution of species richness [34]. This method is based on the principles of geostatistics, enabling the transformation of discrete sample point data into a continuous spatial trend surface while simultaneously providing quantitative information on prediction uncertainty. This provides a reliable and assessable data foundation for the subsequent spatial coupling analysis of carbon stock and recreational services level in this study.Specifically, this study established 56 randomly distributed 10 × 10 m sample in Jinsha Lake Park. Based on the collected plant diversity data, we generated the spatial distribution map using the Ordinary Kriging interpolation method within the GIS geostatistical module. To test the reliability of the interpolation results, we performed cross-validation. ① No apparent systematic bias: The mean prediction error (-0.0699) was close to 0, indicating that the model did not systematically overestimate or underestimate the measured values, and the prediction results were relatively objective. ② Reasonable variance estimation: The standardized root mean square error (1.0378) was close to 1 (the ideal value for Kriging interpolation is 1), indicating that the model’s estimation of error variance was accurate and could reasonably reflect the uncertainty of the predicted values. The generated spatial trend surface can reliably reflect the differentiation pattern of plant diversity (high-medium-low), meeting the requirements for subsequent spatial coupling analysis.

After obtaining the distribution maps for each indicator, the data underwent normalization using the linear function of the “Fuzzy Membership” tool in ArcGIS 10.6. Subsequently, the “Weighted Sum” tool was used to apply the weighted evaluation results as coefficients to derive the overall recreational service level assessment for Jinsha Lake Park.

### Coupling coordination of carbon stock and recreational services level

The concept of coupling was initially used to characterize the interaction mechanisms among multiple circuit components or system modules. Most scholars define coupling as the phenomenon where two or more systems influence each other through various interactions, and it is now widely applied in climate change and environmental research [35]. As dual dimensions of ecosystem services, carbon stock and recreational services level in parks exhibit complex interactive mechanisms. These interrelationships directly influence the overall coordination of urban park systems. Therefore, this study quantitatively assesses the synergistic development level between these two dimensions.


$$f(x)=\sum_{i=\text{1}}^{p}w_{i}x_{i},\;g(y)=\sum_{j=\text{1}}^{m}w_{j}y_{j}$$ (2)

In the formula, f(x) represents the recreational services level evaluation index, and ${\text{w}}_{\text{i}}$ denotes the weight of each recreational services level indicator. Since the assessment of carbon stock itself does not involve multi-indicator weighting, the number of indicators  m=1 and the weight ${\text{w}}_{\text{j}}=\text{1}$,g(y)  is the standardized result of the estimated carbon stock value.


$$\begin{array}{c} C=\frac{\text{2}\sqrt{f(x)\times g(y)}}{f(x)+g(y)}) \end{array}$$ (3)

$$\begin{array}{c} T=\alpha f(x)+\beta g(y) \end{array}$$ (4)

$$\begin{array}{c} D=\sqrt{CT} \end{array}$$ (5)

In the formula, *C* represents the coupling degree, *T* denotes the coordination index, *β* and *α* are undetermined coefficients set to 0.5 in this paper; *D* signifies the coupling coordination degree, with values ranging from 0 to 1. LIAO C B [36] systematically proposed a classification system for the coupling coordination degree, providing corresponding threshold ranges (0-1). This system has been widely adopted and cited by scholars across numerous subsequent fields [37–39]. This study also refers to this classification standard, dividing the coupling coordination degree (D value, where 0.00 ≤ D ≤ 1.00) into 3 major categories and 10 subcategories (see Table 4) to systematically assess the synergistic development level between urban park carbon stock and recreational services level. Specifically, the Coordinated category (0.60 ≤ D ≤ 1.00) indicates that the two systems mutually promote each other and exhibit good synergy. It can be further subdivided into four levels: Primary coordination, Intermediate coordination, Good coordination, and High quality coordination, representing an optimization process from initial synergy to high compatibility. The Transitional category (0.40 ≤ D < 0.60) includes Grudging coordination and On the verge of maladjustment. The system is in a sensitive critical state between coordination and maladjustment, with a tense and fragile relationship, requiring vigilance against degradation risks and proactive optimization. The Maladjusted category (0.00 ≤ D < 0.40) encompasses four levels: Mild maladjustment, Moderate maladjustment, Severe maladjustment, and Extreme maladjustment. This signifies significant constraints or even conflicts between the systems, indicating unsustainable development that urgently requires targeted intervention and systematic improvement. To delve into the root causes of system imbalance under different coordination levels, this study introduced the relative development index (U value) was introduced to determine which system is prioritized and which lags behind, thereby providing a basis for formulating differentiated synergistic enhancement strategies.

**Table 4:** Coupling coordination levels.

Classification	Coupling coordination degree(D value)	Levels	relative development index (U value)	Development Type
Coordinated category (Acceptable range)	0.90-1.00	High quality coordination	0<u≤0.6	Carbon stock lagging
			0.6<u≤1.2	Coordinated development
			u>1.2	Recreational services level lagging
	0.80-0.89	Good coordination	Do.	Carbon stock lagging
				Coordinated development
				Recreational services level lagging
	0.70-0.79	Intermediate coordination	Do.	Carbon stock lagging
				Coordinated development
				Recreational services level lagging
	0.60-0.69	Primary coordination	Do.	Carbon stock lagging
				Coordinated development
				Recreational services level lagging
Transitional category (Transitional range)	0.50-0.59	Grudging coordination	Do.	Carbon stock lagging
				Coordinated development
				Recreational services level lagging
	0.40-0.49	On the verge of maladjustment	Do.	Carbon stock lagging
				Coordinated development
				Recreational services level lagging
Maladjusted category (Unacceptable range)	0.30-0.39	Mild maladjustment	Do.	Carbon stock lagging
				Coordinated development
				Recreational services level lagging
	0.20-0.29	Moderate maladjustment	Do.	Carbon stock lagging
				Coordinated development
				Recreational services level lagging
	0.10-0.19	Severe maladjustment	Do.	Carbon stock lagging
				Coordinated development
				Recreational services level lagging
	0.00-0.09	Extreme maladjustment	Do.	Carbon stock lagging
				Coordinated development
				Recreational services level lagging


$$\begin{array}{c} U=\frac{\text{f}(\text{x})}{\text{g}(\text{y})} \end{array}$$ (6)

In the formula, *U* stands for the level of development, f(x) represents the carbon stock and g(y) represents recreational service level.

## Results and discussions

### Estimate results of carbon stocks

Based on the interpretation of remote sensing imagery and field plot surveys at Jinsha Lake Park, the park’s vegetation overview reveals a total area of 64 hectares. Extensive waterbodies, large-scale structures, and hard surfaces significantly reduce available vegetation space, resulting in relatively limited planting areas. This spatial distribution pattern is clearly confirmed in the NDVI imagery (Fig 3). In terms of community structure and plant composition, the park’s vegetation as a whole exhibits robust growth, and its planting patterns show diversity and spatial differentiation. The west and east zones primarily feature tree-grass structures, while the south area and portions of the northern entrance zones and key nodes feature mixed plantings of tree-shrub-herb and shrub-herb structures. Dominant tree species include *Cinnamomum camphora*, *Ginkgo biloba*, and *Koelreuteria paniculata*, while the shrub layer is mainly composed of *Photinia serratifolia* and *Malus halliana*. Together, these elements form the basic framework of the park’s vegetation.

**Fig 5:**
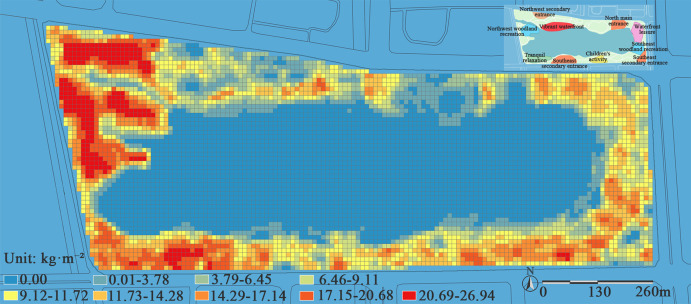
Spatial distribution map of carbon stock in Jinsha Lake Park. The map depicts the simulated carbon stock across the park, with values ranging from 0.00 kg/m^2^ (bare soil/water) to 26.94 kg/m^2^(dense woodland), classified into nine quantile-based intervals represented by a sequential color ramp from blue (low) to red (high). The inset in the upper-right corner provides a reference map of the park’s functional zoning to facilitate the spatial localization and interpretation of specific areas.

The spatial pattern of carbon stock in Jinsha Lake Park exhibits significant heterogeneity, characterized by higher values in the west and south, and lower values in the east and north (Fig 5). A distinct low-carbon stock zone forms within the 20-meter buffer zone surrounding the water body, displaying a layered distribution pattern. The western area of the park, distant from the entrance, is dominated by large tree communities (such as *Cinnamomum camphora* and *Koelreuteria paniculata*), with an average carbon stock reaching up to 26.94 kg/m². In contrast, the northern section, characterized by dense, large commercial buildings and hard-surfaced plazas, exhibits limited vegetation cover, forming a distinct carbon stock depression. This finding further suggests that the spatial overlap between low carbon stock areas and high recreational vitality areas within the park is not coincidental. It essentially reflects a systemic conflict between intensive human activities and ecological functions in limited urban space: the expansion of hardscapes (such as plazas and walkways) to accommodate large crowds and commercial demands often encroaches on vegetation growth space and degrades its community structure, thereby directly undermining core ecological functions such as carbon stock in these areas.

The northwest woodland recreation zone is a high-carbon-stock area with an average carbon density of 16.14 kg/m². Its vegetation structure primarily consists of tall trees complemented by low-growing herbaceous plants. The tree layer is dominated by high-carbon-sequestration species such as *Cinnamomum camphora* and *Koelreuteria paniculata*. The composition of these tree species aligns with the findings of Bhatta et al., whose research confirms that *Cinnamomum camphora* is an excellent high-carbon-sequestering tree species [40]. Furthermore, some preserved native trees exhibit larger DBH and greater age, such as *Cinnamomum camphora*, reaching a DBH of 41 cm and heights of 12 m. In contrast, the average carbon density in the southeast woodland recreation area is only 9.85 kg/m². Although its vegetation structure also primarily consists of a “tree-herb” combination, the core reason for its low carbon stock is the insufficient proportion of high-carbon-sequestering tree species. This finding strongly supports the conclusion by Zhao et al. that plant species selection plays a decisive role in carbon stock [41]. Under the realistic constraint that vegetation coverage or green space area is difficult to increase substantially, scientifically adjusting plant species and their configuration to optimize community structure can significantly enhance the carbon stock per unit area of green space without occupying new land. This approach elevates the “quality” of greening to a position as important as the “quantity” expansion.

The average carbon density in the tranquil relaxation zone and children’s activity zone is 14.17 kg/m² and 11.27 kg/m², respectively. Both areas feature rich vegetation diversity and complex plant community structures, primarily characterized by a typical “tree-shrub-herb” multi-layered structure. In the children’s activity area, to accommodate recreational needs, vegetation is primarily composed of highly ornamental, low-carbon-sequestering plants such as *Prunus serrulata* and *Malus halliana*, resulting in slightly lower carbon stock in this area than in the tranquil relaxation zone.

Additionally, carbon stock varies across the park’s entrance areas: south main entrance: 10.06 kg/m²> northwest secondary entrance: 8.79 kg/m²> southeast secondary entrance: 7.04 kg/m²> north main entrance: 4.94 kg/m². The average carbon density at the main north entrance is the lowest, mainly because large areas of hard paving reduce the space for plant growth.This conclusion aligns with the findings of Li et al., indicating that impervious surfaces and vegetation are the primary factors affecting carbon stock and further confirming the negative correlation between impervious paving and carbon stock [42]. In contrast, the south main entrance combines a plaza with lawn greenery, featuring high carbon-sequestering tree species like *Cinnamomum camphora* and *Acer* spp. Its plant diversity, planting density, and canopy closure significantly exceed those of other entrance areas. These factors collectively contribute to the outh main entrance’s higher carbon density and stronger vegetation carbon sequestration capacity. The gradient differences in carbon stock among various entrances intuitively reflect the impacts of different design concepts on ecological effects. This urges us to re-examine the conventional entrance design paradigm characterized by “open and hardened spaces” and to contemplate how to promote the ecological transformation of entrance spaces in high-intensity-use urban parks, enabling them to become composite nodes that simultaneously possess ecological resilience and social service capacity.

### Evaluation of recreational services level

#### Results of each evaluation index.

**Fig 6:**
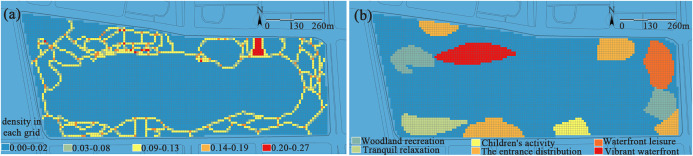
(a) Road accessibility. The road density value is calculated for each grid cell, ranging from 0.00 to 0.27. Visualization uses a red-blue color gradient, where red areas indicate high road density (e.g., plaza and main entrance zones), and a shift toward blue represents gradually decreasing road density. (b) Functional diversity. Based on the park’s master plan and field surveys, and according to the primary recreational activity types it supports, the park is divided into six main functional zones. Different colors in the figure represent distinct functional zones, visually illustrating the spatial distribution of various recreational functions.

(1) **Road network and functional spaces.** Through analyzing the park’s road network as shown in Fig 6(a), it can be observed that the park exhibits a complete gradient of road density (RD), ranging from 0.00 to 0.27. However, the park has insufficient north-south connectivity, which leaves visitors with no alternative routes to turn back when they reach the middle of their journey, they are often forced to retrace their steps or finish the entire route.

From the perspective of functional zones as presented in Fig 6(b), there are significant differences in road density across different areas: the road density in the northern functional zones is much higher than that in the southern ones. Specifically, the entrance distribution area in the north has the highest road density, followed by the vibrant waterfront zone, while the children’s activity area has the lowest. This unbalanced distribution of road density leads to underutilization of spaces in the low-density areas of the western and southern parts of the park. It not only hinders visitors from using various functional spaces conveniently and efficiently but also reduces the park’s overall service quality and visitor experience. As indicated by the research of Chen et al., a well-connected and highly accessible spatial system can significantly enhance the density of human activities [43]. The vitality of various functional zones depends not only on the static provision of facilities but also, more critically, on whether they can be organically linked through an efficient and legible road network. Such integration transforms isolated blocks into an interconnected, mutually supportive, and synergistically operating organic whole.

**Fig 7:**
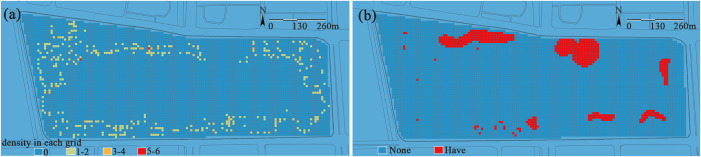
(a) Service facilities. Each grid cell is classified by the number of on-site service facilities (e.g., seats, trash bins, street lamps) it contains. (b) Building and structure distribution. Each grid cell is classified based on the presence or absence of buildings or structures, thereby clearly indicating its spatial locations within the park.

(2) **Service facilities and building/structure distribution.** Analysis of facility distribution shows (Fig 7(a)) that the number of service facilities (including street lamps, trash bins, and seats) per unit area (100 m²) ranges from 1 to 5. Among these, instances with 3–5 facilities within this unit area are rare, and such instances are mainly concentrated along main roads and at key activity venues; most areas only have 1–2 facilities.

Overall, the distribution of facilities is both balanced and reasonable, and it can effectively meet the concentrated needs of visitors. There are several large-scale service buildings in the park as depicted in Fig 7(b), such as the Jinsha Lake Grand Theater in the northeastern part. With a total construction area of approximately 44,000 square meters, the theater integrates cultural exchange, performances and exhibitions, and leisure activities. Its value lies in its ability to attract diverse activities, extend dwell time, and stimulate composite uses in the surrounding area, thereby generating a powerful radiation effect. This radiating force, which amplifies impacts from points to broader areas, serves as a key driver for activating the park’s overall network and continuously enhancing the vitality and attractiveness of the space.

**Fig 8:**
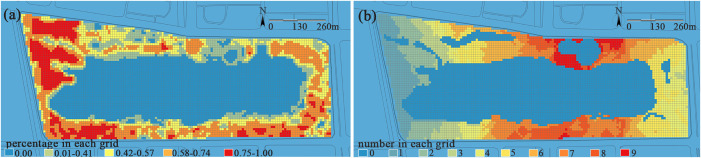
(a) Vegetation coverage. The proportion of each grid cell covered by the vertical projection of vegetation is calculated, with values ranging from 0 to 1. Red indicates areas of high coverage, while blue represents areas of low or no vegetation (e.g., water bodies, hard paving). (b) Plant diversity. The number of plant species recorded within each grid cell ranges from 0 to 9 species. Different colors in the figure represent different classification levels, visually illustrating the spatial variation in plant richness.

(3) **Vegetation.** Analysis of vegetation coverage and plant diversity in the park shows that the two exhibit a significantly opposite spatial distribution pattern: the vegetation coverage in most eastern and western areas reaches a relatively high level of 0.56–1.00, while that in the northern and southern areas is only 0.01–0.55 (Fig 8(a)).

In terms of plant diversity as shown in Fig 8(b), per unit area (100 m²), the number of plant species is the highest in the areas near the main entrance on the northern and southern sides, recorded at 6–9 species or more. These areas are dominated by the compound structure of “tree-shrub-herb”, thus resulting in rich seasonal changes. In contrast, the eastern and western areas have a maximum of 5 plant species within the same unit area, leading to monotonous seasonal changes and insufficient biodiversity. Empirical studies have shown that a sole focus on achieving high vegetation coverage does not guarantee the formation of a rich ecological structure. High-coverage areas are often dominated by a few fast-growing tree species, resulting in simplified species composition and seasonal landscapes. In comparison, areas with high plant diversity, even with relatively limited coverage, can support richer landscape dynamics through their multi-layered “tree–shrub–herb” structure, thereby offering greater advantages in ecological service efficiency per unit area.

**Fig 9:**
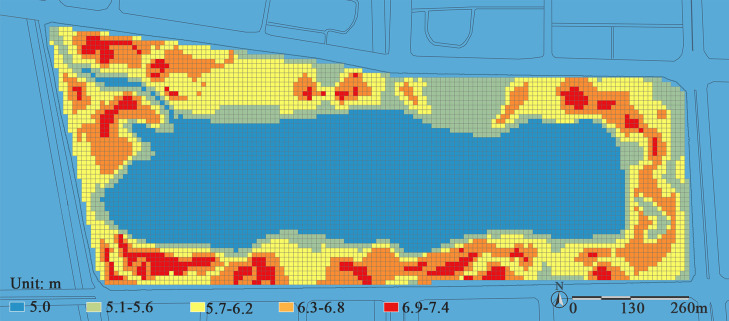
Micro-topography shaping. The topographic height ranges from 5.0 to 7.4 meters. A blue-to-red color gradient is used to represent the transition from low to high elevation, clearly illustrating the micro-relief spatial characteristics of the park’s surface.

**Fig 10:**
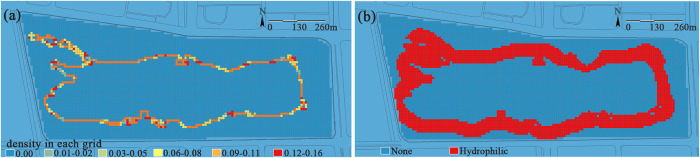
(a) Waterbody morphological. The morphological characteristics are quantified by calculating the sinuosity of the waterbody’s edge and visualized using a blue-red color gradient, where blue represents straight shorelines and red represents sinuous shorelines. (b)Water distribution. The red areas in this figure indicate the waterfront interface zone, representing a critical transition belt with potential hydrophilic functions and ecological influence.

(3) Micro-topography. Jinsha Lake Park features a generally flat topography with a maximum elevation difference of approximately 2 meters, presenting a south-high, north-low gradient (Fig 9). The southern section incorporates micro-topography through plant landscaping, while the northern area, constrained by large-scale building layouts, primarily consists of flat plazas. Topography serves as the fundamental framework of the park ecosystem; the key to ecological construction lies not in large-scale topographic modification, but in the preservation and ingenious utilization of micro-topography. This approach, by leveraging natural variations in spatial form to create an immersive recreational experience, ultimately achieves the dual enhancement of ecological functions and recreational experiences.

(4) Waterbodies. The park’s current shoreline predominantly utilizes soft ecological revetments, creating a winding, meandering profile with expansive waterfront access (Fig 10). However, its limited recreational functions diminish the vitality of water-based activities. This phenomenon reveals a core contradiction in urban waterfront space planning: the inherent tension between ecological preservation and human vitality. While restoring natural processes, ecological shorelines often create physical and psychological barriers—resulting in a condition that can be admired from afar but is difficult to engage with closely for recreation—which can ultimately weaken social vibrancy. The true challenge lies in moving beyond the dichotomy of “preservation versus utilization” and exploring how to skillfully integrate human activities into the ecological framework, thereby achieving an organic integration of ecology and humanity.

### Evaluation of recreational services level

Based on the recreational service evaluation index system constructed in Table 3, this study calculates the overall evaluation of recreational services level at Jinsha Lake Park. Results indicate that recreational services level exhibit a north-south high, east-west low trend. Core nodes such as the vibrant waterfront zone, north and south entrance distribution areas, children’s activity area, and waterfront leisure area demonstrate significantly higher recreational values than the woodland recreation zone and tranquil relaxation zone. Conversely, the northwest and southeast secondary entrance areas represent recreational troughs. This finding indicates that nodes with high recreation values typically exhibit significant spatial associations with main entrances and core scenic areas, where entrance areas undertake initial crowd gathering and functional guidance, while core scenic areas form derivative highlands of supporting services through the radiation of their attractiveness, and this is consistent with the conclusions drawn by Zhang et al. in their study of visitor distribution within theme parks [44].

**Fig 11:**
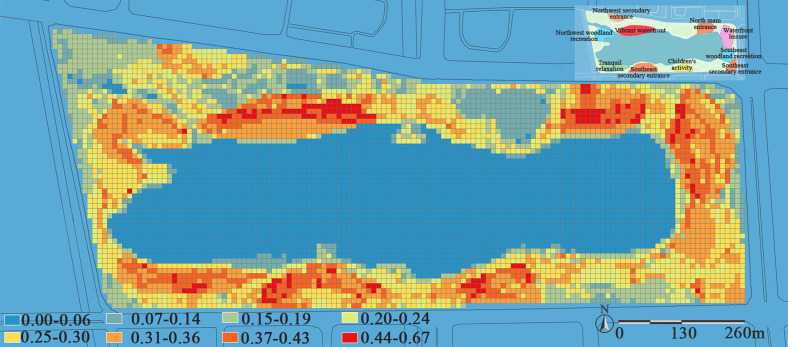
Overall evaluation of recreational services level at Jinsha Lake Park. The recreational service level in the park is measured by a comprehensive index ranging from 0.00 to 0.67 and classified into eight levels. A blue-to-red color gradient is applied for spatial visualization, where blue represents areas with low service levels and red indicates areas with high service levels, effectively revealing the spatial heterogeneity of service provision capacity.

Fig 11 shows that the vibrant waterfront zone achieved the highest overall recreational score (0.390). Located near the park entrance, this area centers on its distinctive man-made beach waterfront landscape as its primary attraction. Combined with excellent transportation accessibility and proximity to the park’s large-scale integrated service buildings, these factors collectively form the core support for its high recreational vitality. However, the insufficient ecological vitality resulting from low plant diversity and sparse vegetation coverage significantly constrains the potential for enhancing overall recreational vitality. This aligns with the findings of Ordóñez et al., revealing a significant positive correlation between vegetation coverage, plant diversity, and recreational satisfaction [45]. This pattern has been further verified in the north-south main entrance areas. Open entrance zones offer excellent spatial accessibility, along with concentrated and comprehensive service facilities, while rich plant diversity further enhances their attractiveness. However, the insufficient overall vegetation coverage (especially arbor canopy density) results in weak shading and cooling functions. The above analysis indicates that a sound vegetation structure significantly influences human comfort, willingness to stay, and psychological restoration through implicit ecological processes such as microclimate regulation and spatial enclosure. In recreational space planning, ecological quality should not be regarded as a “soft” factor that can be replaced by transportation or facility advantages, but rather as a core structural support for the sustainability of recreational vitality.

Children’s activity zone (0.380) enhances site appeal through diverse facility layouts and incorporates varied landscape lighting to improve nighttime activity safety. However, its distance from the main north entrance/Exit distribution area results in weak spatial connectivity. Children’s activities are typically embedded in the context of family outings, encompassing such composite needs as caregiving, rest, and socialization. Therefore, the location selection of children’s activity zones depends not only on the physical distance from the entrance but also, more importantly, on whether they are seamlessly integrated with parental rest areas, service nodes, and the primary activity flow of families. If this behavioral logic is overlooked, even well-equipped children’s activity zones may remain underutilized due to inconvenience, leading to a mismatch characterized by “abundant facilities yet insufficient vitality.”

The tranquil relaxation zone (0.347) and the northwest woodland recreation area (0.343) received relatively lower recreational ratings. The tranquil relaxation zone relies on topography with varying elevations and diverse plant communities, while the woodland recreation area features a single-layer tree canopy structure, creating a space with high canopy closure. An excellent ecological foundation does not automatically translate into high recreational vitality. Although each area possesses distinct natural features in terms of vegetation and topography, their weak road network accessibility and insufficient rest facilities have suppressed visitors’ willingness to enter and the quality of their stay.

The spatial differentiation of park recreation efficiency reveals the inherent limitations of the traditional functional zoning planning approach. While this model clarifies the dominant function of each area, it also tends to make spatial attributes overly singular, hindering the generation of diverse, composite recreational experiences. Therefore, to systematically enhance the overall recreational quality of the park, the planning mindset must shift from static “zoning control” to dynamic “systematic mending.” By promoting functional diversity and spatial interconnectivity, a more resilient and inclusive vitality network can be constructed.

### Coupling coordination analysis

**Fig 12:**
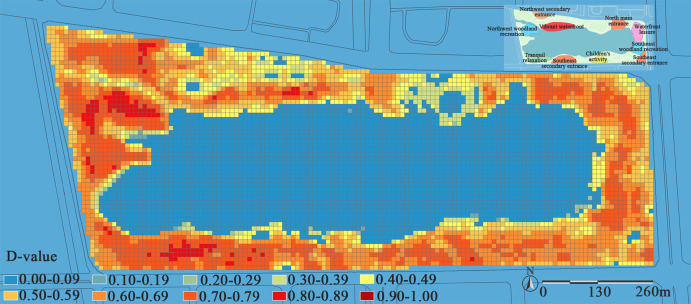
Analysis chart of coupling coordination between carbon stock and recreational services level at Jinsha Lake Park. The figure classifies the coupling coordination degree (D-value, ranging from 0 to 1) into 10 level intervals, each corresponding to a distinct coordination level (detailed in Table 4), and clearly reveals the spatial heterogeneity of the coupling coordination degree across the park.

To analyze the synergy between carbon stock and recreational services level in Jinsha Lake Park, the Feature to Point tool in ArcGIS 10.6 was employed to extract the center point coordinates of fishing nets. A total of 6,321 point locations were obtained and exported to an Excel spreadsheet for analysis of their coupling coordination. The results are shown in Fig 12.

The coupling coordination degree (*D*-value) between carbon stock and recreational services level in Jinsha Lake Park ranges from 0.06 to 0.89 (excluding waterbodies), with most areas falling within the “severe maladjustment” to “good coordination” intervals. The “primary coordination” zone covers the largest area, primarily distributed in the western forest area, the southwestern quiet zone, and parts of the eastern region. “Intermediate coordination” and “on the verge of maladjustment” zones follow, exhibiting a continuous ring-shaped distribution. “Mild maladjustment” and “grudging coordination” zones are concentrated in the northern entrance hub and vibrant waterfront areas, while “high quality coordination” zones are virtually absent. Consequently, enhancing the synergy between carbon stock and recreational services level at Jinsha Lake Park is essential to achieve dual objectives: strengthening ecological conservation and elevating recreational quality.

The area with the highest coupling coordination in Jinsha Lake Park is located in the western section. The woodland recreation zone exhibits a coupling coordination (*D*-value) range of 0.43–0.87, indicating an overall “intermediate coordination” level with *U* = 1.16 (0.60 < *U* < 1.20)(Table 4). The spatial carbon stock and recreational service development level of this area fall within an acceptable “coordinated” range. However, despite possessing an excellent ecological foundation (vegetation, topography, waterbodies, etc.), the area has failed to effectively integrate its native resources to create a multifunctional recreational space combining natural sightseeing and science education. This results in a pattern of “low recreational score – high carbon stock”. Meanwhile, the woodland recreation zone on the eastern side, despite being closer to the main entrance with better accessibility, exhibits a coupling coordination degree (*D*-value) range of 0.41–0.78, indicating only “primary coordination”. The lack of spatial distinctiveness in the region has resulted in limited recreational activities and suboptimal carbon sequestration efficiency. This indicates that viewing road accessibility as the sole determinant of spatial vitality has limitations. While accessibility serves as a fundamental prerequisite for activating spaces, it is the intrinsic qualities of the space itself, unique landscapes, favorable ecological environments, and diverse recreational facilities, that are crucial for achieving high levels of synergistic enhancement in carbon stock and recreational services level.

The highest proportion in park coupling coordination is “primary coordination”. The coupling coordination degree (*D*-value) for children’s activity areas ranges from 0.50 to 0.79, with *U* = 0.74 (0.6 < *U* < 1.2). This indicates that both fall within an acceptable “coordination” range. Combined with previous analyses, this area has limited carbon sequestration capacity due to the lack of high-efficiency carbon-storing tree species. At the same time, its relatively remote location constrains the enhancement of recreational intensity. Both functions are at a moderate level and not particularly prominent; yet they have achieved a coordinated state, which precisely illustrates that a “moderate” level in individual functions does not necessarily lead to systemic dysfunction. On the contrary, as long as the development levels of the two functions are balanced, the system can maintain basic coordination and stability even if neither function performs exceptionally. However, optimization should follow the core principle of “balanced enhancement without mutual hindrance.” Instead of pursuing extreme breakthroughs in individual functions, the focus should be on their synergistic gains and steady and robust improvement.

The park coupling coordination level is “On the verge of maladjustment”, primarily located in the northern area, with the vibrant waterfront zone being a typical example. The coupling coordination degree (*D*-value) ranged from 0.41 to 0.76, with *U* = 0.28 (*U* < 0.6), indicating that carbon storage lags significantly behind recreational services level. This pattern exhibits “high recreational scores–low carbon stock”. This is consistent with the conclusions of existing research: excessive expansion of the built environment in waterfront areas tends to cause an imbalance between ecological and service values [46]. If social service functions develop rapidly while ecological functions fail to keep pace, systemic shortcomings are quickly exposed, directly lowering the overall coordination level. For similar waterfront recreational areas with a *U* value of 0.61, carbon stock lags slightly behind recreational services level, yet both are within an acceptable coordinated range. This suggests that the system has a certain buffering and accommodating capacity. This difference may stem from the moderating effect of “mediating elements” such as vegetation structure, micro-topography, pavement permeability, or activity zoning. A universal coordination pathway is not merely about one-directional compensation, but about enabling ecological functions to positively feed back into the recreational experience—for example, integrating high carbon-sequestering plants with shade-providing rest areas and nature education functions, thereby transforming carbon sequestration capacity into perceptible recreational added value, and ultimately promoting a systemic shift from “competition” to “symbiosis” between the two.

The park coupling coordination level is predominantly “mild maladjustment.” The coupling coordination degree (*D*-value) for the north entrance distribution area ranges from 0.43 to 0.77, with *U* = 0.31 (*U* < 0.6). Its unique waterfront landscape and excellent transportation accessibility contribute to a high recreational score. However, the current planting is sparse and species-limited, lacking a high-carbon-sequestration vegetation layer. This results in a spatial pattern of “high recreational scores–low carbon stock”. This pattern demonstrates that in the main entrance area, the demand for crowd circulation efficiency and symbolic presentation has relegated ecological processes to a secondary position, leading to a maladjusted relationship between the two. In contrast, the south entrance distribution area (U = 0.74) achieves an acceptable level of coordination between the two factors. This indicates that even in spaces subject to high-intensity use, ecology and recreation are not necessarily antagonistic; synergy can be achieved through “small-scale, high-adaptability” ecological design. The underlying logic behind this difference is that spatial coupling relationships are profoundly influenced by their inherent “intrinsic roles”—when a space is assigned a strong, singular social function (such as rapid gathering and dispersal at a main entrance), its internal system is highly prone to developing in a “polarized” manner towards serving that function, while other functions become marginalized. Therefore, grasping the inherent characteristics of different space types is key to assessing their coordination status.

Therefore, Jinsha Lake Park exhibits distinct spatial characteristics, specifically “high recreational level–low carbon stock” and “low recreational level–high carbon stock.” An urban park is not a homogeneous space; the ecological and recreational functions of its various internal areas often differ significantly. Therefore, promoting their synergistic development should not pursue a uniform standard. Instead, based on understanding the specific characteristics and conditions of different areas, appropriate, concrete, and refined enhancement approaches should be adopted.

## Implications

### Optimize functional zoning

Research findings indicate that park accessibility serves as a fundamental prerequisite for activating spatial functions. The functional spaces of parks determine the intrinsic attributes of their internal spaces, such as vegetation structure and facility types. Only through the rational allocation of functional zones can high-level synergistic enhancement between carbon stock level and recreational services level be achieved.

The empirical results of this study indicate that the ecological function zones within Jinsha Lake Park generally exhibit a “low recreational level–high carbon stock” distribution pattern. For areas in urban parks that are “ecologically strong but lack recreational vitality” (such as wilderness woodlands and ecological wetlands), the principle of “conservation priority, experience integration” should be followed. The goal is to activate their recreational value while strictly protecting their high carbon stock baseline. This can be achieved through minimal facility interventions, such as establishing ecological trails, observation points, and educational signage, thereby transforming the carbon sequestration process into a tangible natural experience. This approach avoids the two extremes of “over-protection leading to idle value” and “over-development damaging the ecological core.”

For hard activity spaces with high-intensity use—characterized by “high recreational level–low carbon stock” (such as main entrance plazas and core gathering areas)—renewal does not require large-scale demolition and reconstruction. Instead, a “small-scale, precision-oriented” ecological integration strategy should be adopted. This involves embedding modular green islands within paved plazas, arranging mobile tree planters with high carbon-sequestering trees at dispersal nodes, and creating multi-layered plant communities through micro-topography shaping. This strategy essentially follows a design logic of “spatial fragmentation–ecological integration,” transforming single-function hardscapes into a composite system where “dispersal modules” and “ecological modules” are nested within each other. This ensures pedestrian efficiency and activity capacity while effectively embedding carbon stock carriers.

### Optimize the layout of plant landscapes

Optimizing vegetation configuration is central to synergistically enhancing carbon stock and recreational services. A well-designed plant arrangement not only directly increases regional carbon stock but also enhances recreational value through its coverage and diversity. Design can incorporate differentiated planting structures tailored to specific landscape space requirements. Regardless of park type or scale, vegetation configuration should adhere to the core principle of “aligning carbon stock with recreational demand” and adopt differentiated designs for different functional spaces. Specifically: Open activity areas should emphasize shade provision and open sightlines by planting broad‑canopy trees with high carbon-sequestration capacity, thereby translating their carbon-sequestration potential into tangible cooling and recreational benefits. Ecological zones should emphasize high carbon stock and community structural richness. This can be achieved by preserving and supplementing native vegetation with high carbon-sequestration potential and establishing low-impact eco-touring routes, thereby simultaneously enhancing ecological awareness and aesthetic experience. In terms of species selection, priority should be given to highly adaptable native tree species with high carbon-sequestration capacity (i.e., high-carbon-sequestering native tree species). Such species offer stable contributions to carbon stock, can reduce maintenance costs, and help mitigate the risk of invasive species introduction. In summary, skillfully integrating carbon stock enhancement with recreational experience optimization in vegetation configuration is a key pathway to achieving synergistic improvement of ecological and recreational functions.

## Conclusions

### Conclusions

From a systems perspective, this study proposes an integrated research framework for the multifunctional analysis of urban parks. This framework identifies the synergistic relationship between park carbon stock and recreational services level, and outlines optimization strategies to achieve the dual objectives of ecological conservation and enhanced recreational quality.

(1) Results indicate that the coupling coordination degree between carbon stock and recreational services level in Jinsha Lake Park exhibits a distribution pattern characterized by higher values in the west and lower values in the east, as well as higher values in the south and lower values in the north. Most of these coupling coordination degrees fall in the “primary coordination” range and exhibit distinct spatial characteristics such as “high recreational level – low carbon stock” and “low recreational level – high carbon stock,” indicating that the park needs to further enhance the synergy between its carbon stock and recreational services level.
(2) Optimizing vegetation configuration serves as the core engine for synergistically enhancing carbon stock and recreational services level. A rational plant arrangement not only directly strengthens regional carbon stock, but vegetation coverage and plant diversity also stimulate recreational vitality. For instance, coordinating the proportion of high carbon-sequestering tree species with ornamental species can directly and efficiently enhance both carbon sink capacity and recreational vitality simultaneously.
(3) Park accessibility is a fundamental prerequisite for activating the park space, while functional zones determine the intrinsic attributes of its internal areas, such as vegetation structure and facility types. The rational configuration of functional zones is a critical factor influencing the synergy between a park’s carbon stock and its recreational services level.

### Limitations

This study has certain limitations. (1) The indicators for evaluating recreational services level require further refinement. Currently, the assessment primarily relies on objective metrics from the perspective of park designers and builders. Future research should collect more foundational data to conduct a more comprehensive and detailed evaluation of recreational activity levels. (2) This study focuses primarily on above-ground vegetation carbon sinks, excluding below-ground carbon sinks such as soil carbon pools and litter. Future research should incorporate more comprehensive carbon stock accounting to fully reveal its functional characteristics. (3) The study site is Jinsha Lake Park, where representative analysis points with significant variations were selected. Future work will assess the coupling coordination levels and respective development stages of carbon stock and recreational functions across homogeneous functional zones in multiple parks. This will enable the adoption of configuration models from high-coordination parks of similar types to guide spatial optimization design.

## References

[1] Dulal HB, Akbar S. Greenhouse gas emission reduction options for cities: Finding the “Coincidence of Agendas” between local priorities and climate change mitigation objectives. Habitat International. 2013;38:100–5. doi: 10.1016/j.habitatint.2012.05.001

[2] Yu X, Wu Z, Zheng H, Li M, Tan T. How urban agglomeration improve the emission efficiency？A spatial econometric analysis of the Yangtze River Delta urban agglomeration in China. J Environ Manage. 2020;260:110061. doi: 10.1016/j.jenvman.2019.110061 32090809

[3] Holtz G, Xia-Bauer C, Roelfes M, Schüle R, Vallentin D, Martens L. Competences of local and regional urban governance actors to support low-carbon transitions: Development of a framework and its application to a case-study. Journal of Cleaner Production. 2018;177:846–56. doi: 10.1016/j.jclepro.2017.12.137

[4] Arzberger S, Egerer M, Suda M, Annighöfer P. Thermal regulation potential of urban green spaces in a changing climate: Winter insights. Urban Forestry & Urban Greening. 2024;100:128488. doi: 10.1016/j.ufug.2024.128488

[5] Feng L, Wang J, Liu B, Hu F, Hong X, Wang W. Does Urban Green Space Pattern Affect Green Space Noise Reduction? Forests. 2024;15(10). doi: 10.3390/f15101719

[6] Vieira J, Matos P, Mexia T, Silva P, Lopes N, Freitas C, et al. Green spaces are not all the same for the provision of air purification and climate regulation services: The case of urban parks. Environ Res. 2018;160:306–13. doi: 10.1016/j.envres.2017.10.006 29040950

[7] Min K. Exploring Research Fields in Green Buildings and Urban Green Spaces for Carbon-Neutral City Development. Buildings. 2025;15(9):1463. doi: 10.3390/buildings15091463

[8] Larson LR, Jennings V, Cloutier SA. Public Parks and Wellbeing in Urban Areas of the United States. PLoS One. 2016;11(4):e0153211. doi: 10.1371/journal.pone.0153211 27054887 PMC4824524

[9] Liu Y, Xia C, Ou X, Lv Y, Ai X, Pan R, et al. Quantitative structure and spatial pattern optimization of urban green space from the perspective of carbon balance: A case study in Beijing, China. Ecological Indicators. 2023;148:110034. doi: 10.1016/j.ecolind.2023.110034

[10] Wang R-Y, Mo X, Ji H, Zhu Z, Wang Y-S, Bao Z, et al. Comparison of the CASA and InVEST models’ effects for estimating spatiotemporal differences in carbon storage of green spaces in megacities. Sci Rep. 2024;14(1):5456. doi: 10.1038/s41598-024-55858-0 38443413 PMC10914835

[11] Jin X, Lin S, Zhu J, Tan FL, Zhang HG, Chen QC, et al. Dominant tree species and their age groups drive forest carbon storage in Wuyi Mountain National Park, China. Forests. 2024;15(3). doi: 10.3390/f15030546

[12] Moon T, Kim M, Chon J. Adaptive green space management strategies for sustainable carbon sink parks. Urban Forestry & Urban Greening. 2024;94:128236. doi: 10.1016/j.ufug.2024.128236

[13] Li X, Jiang Y, Liu Y, Sun Y, Li C. The impact of landscape spatial morphology on green carbon sink in the urban riverfront area. Cities. 2024;148:104919. doi: 10.1016/j.cities.2024.104919

[14] Dimobe K, Kuyah S, Dabré Z, Ouédraogo A, Thiombiano A. Diversity-carbon stock relationship across vegetation types in W National park in Burkina Faso. Forest Ecology and Management. 2019;438:243–54. doi: 10.1016/j.foreco.2019.02.027

[15] Ali S, Khan SM, Siddiq Z, Ahmad Z, Ahmad KS, Abdullah A, et al. Carbon sequestration potential of reserve forests present in the protected Margalla Hills National Park. Journal of King Saud University - Science. 2022;34(4):101978. doi: 10.1016/j.jksus.2022.101978

[16] Cheng F, Ou G, Wang M, Liu C. Remote Sensing Estimation of Forest Carbon Stock Based on Machine Learning Algorithms. Forests. 2024;15(4):681. doi: 10.3390/f15040681

[17] Oehmcke S, Li L, Trepekli K, Revenga JC, Nord-Larsen T, Gieseke F, et al. Deep point cloud regression for above-ground forest biomass estimation from airborne LiDAR. Remote Sensing of Environment. 2024;302:113968. doi: 10.1016/j.rse.2023.113968

[18] Zhang R, Peng S, Sun F, Deng L, Che Y. Assessing the social equity of urban parks: An improved index integrating multiple quality dimensions and service accessibility. Cities. 2022;129:103839. doi: 10.1016/j.cities.2022.103839

[19] Hamstead ZA, Fisher D, Ilieva RT, Wood SA, McPhearson T, Kremer P. Geolocated social media as a rapid indicator of park visitation and equitable park access. Computers, Environment and Urban Systems. 2018;72:38–50. doi: 10.1016/j.compenvurbsys.2018.01.007

[20] OTHMAN R. THE INFLUENCE OF URBAN PARK GREEN SPACES, PLANT MATERIAL SPECIFICATIONS AND SPATIAL DESIGN ORGANIZATION AND PATTERN TOWARDS CARBON SEQUESTRATION RATE. Appl Ecol Env Res. 2019;17(4). doi: 10.15666/aeer/1704_80798088

[21] Liu D, Li H, Qiu M, Liu Y. Understanding coupled coordination relationships between social and ecological functions of urban green spaces. Geo-spatial Information Science. 2022;26(3):431–45. doi: 10.1080/10095020.2022.2134057

[22] Zhao Y, Guo X, Zhong LH, Wang J, Chen JD. Wall-to-wall above-ground biomass estimation with ALOS-2 PALSAR-2 L-band SAR data and GEDI. IEEE. 2023;:3318–21.

[23] Determining the Effects of Changes in Land Use on Carbon Amount in Above-Ground Biomass with NDVI. Global NEST Journal. 2022. doi: 10.30955/gnj.004542

[24] Li N, Deng L, Yan G, Cao M, Cui Y. Estimation for Refined Carbon Storage of Urban Green Space and Minimum Spatial Mapping Scale in a Plain City of China. Remote Sensing. 2024;16(2):217. doi: 10.3390/rs16020217

[25] He HZ, Huang LH, Duan X, He RK. Study on biomass in main afforestation tree species of the second ring forest-belt of Guiyang. Guizhou Science. 2007;(3):33–9.

[26] Wang C. Biomass allometric equations for 10 co-occurring tree species in Chinese temperate forests. Forest Ecology and Management. 2006;222(1–3):9–16. doi: 10.1016/j.foreco.2005.10.074

[27] Jo H-K. Impacts of urban greenspace on offsetting carbon emissions for middle Korea. J Environ Manage. 2002;64(2):115–26. doi: 10.1006/jema.2001.0491 11995235

[28] Warwick-Champion E, Davies KP, Barber P, Hardy N, Bruce E. Characterising the Aboveground Carbon Content of Saltmarsh in Jervis Bay, NSW, Using ArborCam and PlanetScope. Remote Sensing. 2022;14(8):1782. doi: 10.3390/rs14081782

[29] Donahue ML, Keeler BL, Wood SA, Fisher DM, Hamstead ZA, McPhearson T. Using social media to understand drivers of urban park visitation in the Twin Cities, MN. Landscape and Urban Planning. 2018;175:1–10. doi: 10.1016/j.landurbplan.2018.02.006

[30] Hao J, Gao T, Qiu L. How do species richness and colour diversity of plants affect public perception, preference and sense of restoration in urban green spaces?. Urban Forestry & Urban Greening. 2024;100. doi: 10.1016/j.ufug.2024.128487

[31] Wang Y, Shi X, Cheng K, Zhang J, Chang Q. How do urban park features affect cultural ecosystem services: Quantified evidence for design practices. Urban Forestry & Urban Greening. 2022;76:127713. doi: 10.1016/j.ufug.2022.127713

[32] Franek J, Kresta A. Judgment scales and consistency measure in AHP. 2014;:164–73.

[33] Peng X, Mohamed Afla MR. A Multi-Dimensional Assessment of Pocket Park Landscapes: Insights from Scenic Beauty Estimation and Analytic Hierarchy Process in Dadukou District, Chongqing. Sustainability. 2025;17(5):2020. doi: 10.3390/su17052020

[34] Liu C, Xing SH, Yao Y, Zhang HX, Wang XK. Spatial distribution pattern and influencing factors of spontaneous plants within the built-up areas of Beijing, China. Acta Ecologica Sinica. 2024;44(2):544–58. doi: 10.20103/j.stxb.202205131344

[35] Li Y, Li Y, Zhou Y, Shi Y, Zhu X. Investigation of a coupling model of coordination between urbanization and the environment. J Environ Manage. 2012;98:127–33. doi: 10.1016/j.jenvman.2011.12.025 22265813

[36] LIAO CB. Quantitative judgement and classification system for coordinated development of environment and economy—A case study of the city group in the Pearl River Delta. Tropical Geography. 1999;(2):76–82. doi: 10.13284/j.cnki.rddl.000443

[37] Zhang Z, Hao M, Mao Y, Chen S. The Role of Campus Green Space for Residents: Based on Supply–Demand of Recreation Services. Sustainability. 2024;16(16):6997. doi: 10.3390/su16166997

[38] Li Z, Hu L, Lin A, Chen J, Xu Y. The greener, the richer, the happier?——Spatial distribution and coupling analysis of urban green space and residents’ emotion based on social media data. Ecological Indicators. 2025;177:113754. doi: 10.1016/j.ecolind.2025.113754

[39] Yu Y, Yang X, Li X, Qian L, Zhou S. Supply-Demand Matching Evaluation and Coupling Coordination of Cultural Ecosystem Services in Urban Park Green Spaces. LA. 2025;32(3):90–9. doi: 10.3724/j.fjyl.202408260483

[40] Bhatta SP, Sharma KP, Balami S. Variation in Carbon Storage Among Tree Species in The Planted Forest of Kathmandu, Central Nepal. Current Science. 2018;115(2):274. doi: 10.18520/cs/v115/i2/274-282

[41] Zhao D, Cai J, Xu Y, Liu Y, Yao M. Carbon sinks in urban public green spaces under carbon neutrality: A bibliometric analysis and systematic literature review. Urban Forestry & Urban Greening. 2023;86:128037. doi: 10.1016/j.ufug.2023.128037

[42] Li Y, Yang X, Wu B, Zhao J, Jiang W, Feng X, et al. Spatio-temporal evolution and prediction of carbon storage in Kunming based on PLUS and InVEST models. PeerJ. 2023;11:e15285. doi: 10.7717/peerj.15285 37250707 PMC10215775

[43] Chen J, Tao Z, Wu W, Wang L, Chen D. Influence of Urban Park Pathway Features on the Density and Intensity of Walking and Running Activities: A Case Study of Shanghai City. Land. 2024;13(2):156. doi: 10.3390/land13020156

[44] Zhang YS, Li X, Su Q. Does spatial layout matter to theme park tourism carrying capacity?. Tourism Management. 2017;61:82–95. doi: 10.1016/j.tourman.2017.01.020

[45] Ordóñez C, Labib SM, Chung L, Conway TM. Satisfaction with urban trees associates with tree canopy cover and tree visibility around the home. NPJ Urban Sustain. 2023;3(1):37. doi: 10.1038/s42949-023-00119-8 38666053 PMC11041773

[46] Xiong S, Yang F, Gu C. Exploring spatiotemporal coupling effects between built environment and ecosystem health: Toward static–dynamic sustainable management in waterfront cities. Journal of Cleaner Production. 2025;503:145389. doi: 10.1016/j.jclepro.2025.145389

